# Improving the reliability of the iron concentration quantification for iron oxide nanoparticle suspensions: a two-institutions study

**DOI:** 10.1007/s00216-018-1463-2

**Published:** 2018-11-12

**Authors:** Rocio Costo, David Heinke, Cordula Grüttner, Fritz Westphal, M. Puerto Morales, S. Veintemillas-Verdaguer, Nicole Gehrke

**Affiliations:** 1 Instituto de Ciencia de Materiales de Madrid (ICMM), Consejo Superior de Investigaciones Científicas (CSIC), Sor Juana Inés de la Cruz 3, Cantoblanco, 28049 Madrid, Spain; 2 NanoPET Pharma GmbH, Robert-Koch-Platz 4, Luisencarrée, 10115 Berlin, Germany; 3 Micromod Partikeltechnologie GmbH, Friedrich-Barnewitz-Str. 4, 18119 Rostock, Germany

**Keywords:** Iron analyses, Iron oxide nanoparticles, Colorimetric analysis, Inductively coupled plasma analysis, Comparative study, Inter-institutional study

## Abstract

Most iron oxide nanoparticles applications, and in special biomedical applications, require the accurate determination of iron content as the determination of particle properties from measurements in dispersions is strongly dependent on it. Inductively coupled plasma (ICP) and spectrophotometry are two typical worldwide used analytical methods for iron concentration determination. In both techniques, precise determination of iron is not straightforward and nanoparticle digestion and dilution procedures are needed prior to analysis. The sample preparation protocol has been shown to be as important as the analytical method when accuracy is aimed as many puzzling reported results in magnetic, colloidal, and structural properties are simply attributable to inadequate dissolution procedures. Therefore, a standard sample preparation protocol is needed to ensure the adequate and complete iron oxide nanoparticle dissolution and to harmonize this procedure. In this work, an interlaboratory evaluation of an optimized iron oxide nanoparticle digestion/dilution protocol was carried out. The presented protocol is simple, inexpensive, and does not involve any special device (as microwave, ultrasound, or other high-priced digestion devices). Then, iron concentration was measured by ICP-OES (performed in ICMM/CSIC-Spain) and spectrophotometry (NanoPET-Germany) and the obtained concentration values were analyzed to determine the most probable error causes. Uncertainty values as low as 1.5% were achieved after the optimized method was applied. Moreover, this article provides a list of recommendations to significantly reduce uncertainty in both sample preparation and analysis procedures.

**Figure:**
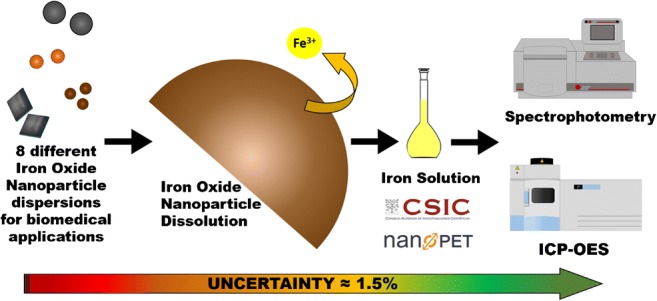
Graphical abstract

Graphical abstract

## Introduction

Iron oxide nanoparticles are widely used in ex-vivo bioassays for the detection and separation of small molecules, biomarkers, DNA, and bacteria [1], and also in-vivo medical diagnosis to enhance the contrast of magnetic resonance imaging (MRI) [2]. In cancer therapies, they have increased the efficiency of different drugs when used as delivery platforms assisted by magnetic fields to concentrate the drug or heat in the targeted tumor area [3]. They are also promising tools for gene therapy and tissue regeneration, among other applications in the biomedical field [4]. Other emerging applications of these nanoparticles include their use in catalysis, and as adsorbents or reactive agents in environmental applications [5]. Typically, magnetic nanoparticles are dispersed in aqueous media to form stable colloidal suspensions, which are commonly studied with a multitude of characterization techniques, focusing on structural, colloidal, and magnetic properties [6]. Many of these properties are strongly related to the concentration or overall amount of iron present in the suspension. Thus, the accurate determination of the iron concentration is crucial for the precise determination of physical and chemical parameters of the iron oxide nanoparticles, e.g., the saturation magnetization in Am^2^/Kg Fe, the specific absorption rate (SAR) in W/g Fe, or the concentration of functional groups on the particle surface in μmol/g Fe, respectively.

Up to our knowledge,there is only one published protocol for the determination of iron in magnetic nanoparticles for biomedical applications using spectrophotometry [7]. In several other applications where iron had to be determined in complex biological materials [8], food [9, 10], and pharmaceutical products [11, 12], the uncertainties of the iron determination among different methods were tested. The current development of the standard ISO/DTS 19807-1 for the magnetic nanosuspensions highlighted the outmost relevance of the determination of the iron content by inductively coupled plasma-optical emission spectroscopy (ICP-OES) and inductively coupled plasma mass spectrometry (ICP-MS) [13].

Particularly in the case of iron oxide nanoparticle suspensions, the sample preparation procedure poses some “pitfalls” for example incorrect pipetting of viscous colloids or incomplete digestion of coated nanoparticles, and contributes significantly to the uncertainty of the obtained concentration values [14]. This might be one reason why our personal experience as well as many discussion with colleagues who work with magnetic nanoparticles show us that concentration values provided for iron oxide nanoparticle suspensions are not reliable in case of both, research samples as well as commercially available iron oxide nanoparticle suspensions, and was the motivation of our study. This type of problems has been identified previously for a variety of inorganic nanoparticles [15]. Here, the quantification of the iron concentration of various iron oxide nanoparticle suspensions was performed in two different labs with two different established methods with the aim to explore and minimize the uncertainty of the obtained results.

First of all, we selected iron oxide nanoparticle suspensions synthesized by different methods, in organic and aqueous media and coated with different materials, namely either small highly charged molecules such as dimercaptosuccinic acid, polysaccharide-based compounds with low charge and high steric repulsion such as dextran and starch or synthetic polymers like polystyrene or poly(acrylic acid)/polyacrylate. The iron oxide core size of the multi-core iron oxide particles varied from about 7 to 28 nm. The highly charged particles with small cores in the range of 7 to 11 nm have been proposed as contrast agents for MRI [16] while the larger dextran-coated particles with core diameters of about 28 nm are ideal for magnetic hyperthermia treatments [17]. On the other hand, the particles encapsulated in a synthetic polymer matrix are interesting tools for bioassays because of their large magnetic moment, long-term stability, and presence of functional groups as basis for conjugation of biomolecules [18].

Secondly, we used widely applied methods for the determination of the iron concentration of aqueous particle suspensions such as ICP-OES and spectrophotometry. For both methods, the magnetic nanoparticles have to be dissolved by highly concentrated acid to release the iron ions into solution. ICP-OES is used for highly accurate and sensitive analysis of major and minor chemical elements in a wide range of materials [19]. It involves introducing a sample solution into the core of high temperature inductively coupled argon plasma (ICP). At temperatures near 8000 °C, the thermally excited elements emit light at characteristic wavelengths, which is detected by a spectrometer. The light is diffracted, amplified, and analyzed to identify and quantify elemental concentrations in the sample. The intensity of the emission is indicative of the concentration of the element within the sample. With the aid of calibration values obtained from suitable standards of known concentration, the content of a certain compound in an unknown sample can be calculated. In case of spectrophotometry, the Fe^3+^ ions are quantitatively reduced to Fe^2+^ by an excess of hydroxylamine hydrochloride (NH_2_OH·HCl). After reaction with a suitable chelator, a colored complex with a characteristic absorption maximum is formed. The iron concentration values are then calculated by means of a calibration curve obtained from iron standard solutions.

Finally, an inter-institutional study has been performed in this work, including the preparation of the sample for the analysis and the validation and harmonization of the iron concentration determination procedures via ICP-OES and spectrophotometry in order to obtain consistent and reliable iron concentration values. We have determined and minimized the uncertainty of the obtained values and a list of recommendations for keeping the uncertainty below 3% has been generated. Special attention was paid to the sample preparation, i.e., digestion and dilution process, which is a crucial step for the iron quantification by the two chosen experimental techniques.

## Materials and methods

### Magnetic nanoparticle suspensions

The samples involved in the cross-validation experiments were magnetic iron oxide aqueous suspensions bearing nanoparticles with hydrodynamic diameters between 70 and 160 nm. They were prepared following different methods previously described and are summarized in Table 1.

**Table 1:** Aqueous iron oxide nanoparticle samples with hydrodynamic diameters in the range of 70 to 160 nm prepared by different methods

Sample	Preparation method	Core size (nm)	PDI σ/D_TEM_	Coating	Hydrodynamic size (nm)	PDI σ/D_HYD_
O1@DMSA	Thermal decomposition in organic media	10	0.08	meso-2,3 Dimercaptosuccinic acid	98 ± 1	0.46
O2@DMSA		7	0.13		161 ± 2	0.6
W1@D	Oxidative precipitation in aqueous media	25	0.25	Dextran	72 ± 1	0.3
W2@D		28	0.2		108 ± 1	0.3
R1@PAA	Borohydride reduction	7.5	0.18	Poly(acrylic acid)	72 ± 1	0.5
R1@PS				Polystyrene	131 ± 1	0.36
HPH1@ST	High-pressure homogenization	25	0.29	Starch	115 ± 1	0.28
HPH1@PA				Poly(acrylate)	126 ± 1	0.35

In brief, magnetic iron oxide nanoparticle samples O1@DMSA and O2@DMSA were obtained by thermal decomposition of iron oleate in organic media [20]*.* These particles were transferred from organic medium to water at pH 7 through a ligands exchange process where oleic acid is slowly substituted by meso-2,3-dimercaptosuccinic acid (DMSA) [21]. The suspensions have hydrodynamic diameter of 98 and 161 nm for O1@DMSA and O2@DMSA, respectively. The amount of coating onto the particle surface was determined by thermal analysis and is similar in both systems (16 and 12% for O1@DMSA and O2@DMSA, respectively) being the magnetic core size the main difference between them. Small iron oxide nanoparticles with an mean core size of 7.5 nm were also produced by reduction of aqueous salts of iron (III) using sodium borohydride followed by surface modification with poly(acrylic acid) (samples R1@PAA) [22]. The same cores were encapsulated into polystyrene/poly(styrene-alt-maleic) acid spheres (R2@PS) by a solvent evaporation method [22] obtaining polymer capsules with a hydrodynamic diameter of 131 nm.

Larger cubic-shaped nanoparticles 25 and 28 nm of core size were synthesized by an aqueous route starting from Fe(II) salt and controlling oxidation with a mild oxidant such as potassium nitrate [23]. An acid treatment [24] was used to oxidize magnetite to maghemite activating the surface and subsequently, the particles were coated with dextran under high-pressure homogenization (HPH) [25] and fractionated in two samples W1@D and W2@D, with hydrodynamic diameters of 72 and 108 nm, respectively and a proportion of dextran of 43% wt determined by thermal analysis. Finally, iron oxide cores of 25 nm were also prepared under high-pressure homogenization conditions (HPH1@ST and HPH1@PA) and coated with starch and sodium poly(acrylate) (MW 1200 Da) respectively by the same method, resulting in particles with hydrodynamic diameters of 115 and 126 nm, respectively. The homogeneity of the particle size was achieved by magnetic fractionation by using a SEPMAG-Q100 [26].

Particle core size was determined by transmission electron microscopy (TEM) using two different apparatus: a JEOL JEM-200 FX microscope operated at 200 keV and a JEOL JEM1010 microscope operated at 100 kV. TEM samples were prepared by placing one drop of a dilute particle suspension on an amorphous carbon-coated copper grid and evaporating the solvent at room temperature. The mean core size of each sample was calculated by measuring the largest internal dimension of at least 100 particles. Polydispersity index (PDI) was calculated as the ratio of the standard deviation and the average core diameter [26].

A Zetasizer Nano-ZS90 from Malvern Instruments was used to determine the hydrodynamic diameter (Z average value in intensity) and polidispersity index of the samples by measuring the dynamic light scattering of suspensions at around 0.5 M Fe concentration. The polidispersity values obtained from the DLS measurements are in fact (σ/Z_ave_)^2^ [27], so for the comparison with the PDI values obtained by TEM the square root of the DLS reported polidispersity index is presented in Table 1. Simultaneous TG and DTA analysis were performed in a Seiko TG/DTA 320U thermobalance. Samples were heated from room temperature to 700 °C at 10 °C/min under an air flow of 100 mL/min. Platinum pans were used and α-Al_2_O_3_ was used as reference.

### Sample preparation for ICP and spectrophotometry

One of the key steps in the analytical process prior to the actual iron detection/quantification is the complete dissolution of the iron oxide nanoparticles, including the quantitative release of the iron ions into solution. This step also serves the dilution of the sample to adjust the sample concentration into the range suitable for detection (1–20 ppm Fe for ICP-OES or 45–358 μM Fe (2.5–20 ppm Fe) for spectrophotometry).

**Fig. 1:**
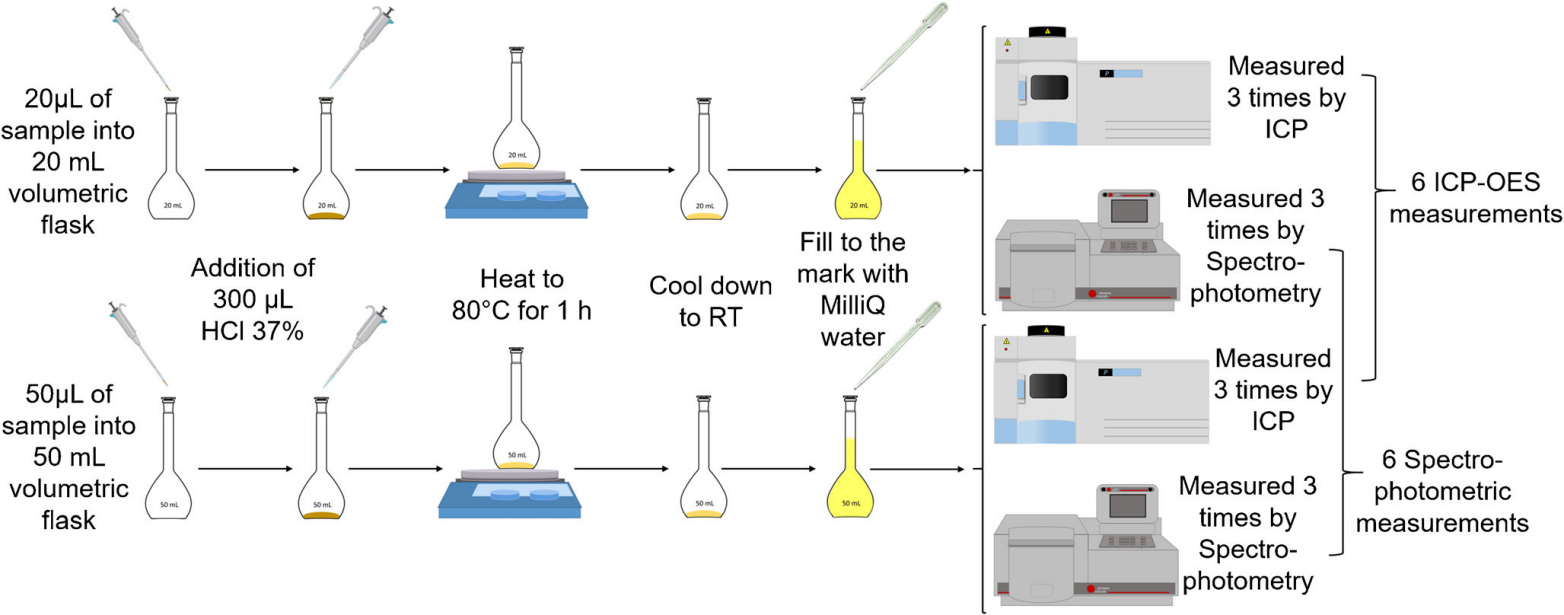
Graphical representation of the steps of the optimized protocol for particle preparation, including digestion and dilution: 20/50 μL of the sample were pipetted into a 20/50 mL volumetric flask. Then, 300 μL HCl 37% were added. The mixtures were heated to approximately 80 °C for one hour. After this time, the solutions were allowed to cool down to RT and the flasks were subsequently filled up to the mark with MilliQ water resulting in two individual dilutions for each sample, which were measured three times either by ICP-OES or by photometry. As a result, we obtained six iron concentration values for each sample which have been used for the calculation of the average iron concentration

To ensure consistent analysis procedures, the number of dilutions prepared per sample and the number of repetitive measurements of each dilution were standardized: each sample was digested and diluted twice using different sample and flask volumes and each dilution was measured three times, resulting in six absorption (and thus, concentration) values per sample in total for each analysis, from which the mean value and the standard deviation were calculated Fig. 1.

For an appropriate sample preparation, the respective sample’s iron concentration was roughly estimated based on the iron concentration communicated by the institution providing the sample. In case of unknown samples, this is done by comparison of the color intensity with known iron oxide nanoparticle references. Based on this estimation, the sample was dissolved using concentrated hydrochloric acid and diluted to adjust its iron concentration approximately into the middle of the calibration line (~ 150 μM Fe).

The following example describes this procedure for a sample with an initial iron concentration of about 150 mM Fe: 20 μL of the sample were pipetted (with a 2–20 μL variable volume Eppendorf research® pipette) into a 20 mL volumetric flask (DURAN® volumetric flask, class A). Then, 300 μL HCl 37% were added. Another 50 μL of the sample were transferred (with a 10–100 μL variable volume Eppendorf research® pipette) into a 50 mL volumetric flask (DURAN® volumetric flask, class A) and 300 μL HCl 37% were added. To assure complete dissolution, the mixtures were heated to approximately 80 °C for 1 h. After this time, the solutions were allowed to cool down to room temperature and the flasks were subsequently filled up to the mark with MilliQ water resulting in two individual dilutions for each sample.

### Standards

For the quantification of the iron concentration with ICP-OES, a calibration line was generated by four standard samples containing between 1 and 20 ppm Fe. The standard samples were prepared by diluting an Iron Plasma Emission Standard (ICP-MS) ICP-MS-27N-0.01 X-1 from AccuStandard® to the respective concentrations by serial dilution.

For the spectrophotometric quantification of the iron concentration, a calibration curve was generated using four iron standards with concentrations between 45 and 358 μM Fe (2.5–20 ppm Fe). These standards were prepared by diluting a Titrisol® Iron Standard (1000 mg Fe, FeCl_3_ in 15% HCl) from MERCK® filled up to 1000 mL MilliQ water in a 1000 mL volumetric flask (DURAN® volumetric flask, class A) to the respective concentrations by a dilution series.

### Measurement of iron content by ICP-OES

ICP-OES (Optima 2100 DV, Perkin Elmer Instruments, Shelton, CT, USA) was carried out with operating parameters shown in Table 2. The wavelength used for iron determination was 238.204 nm but an extra wavelength was used (239.562 nm) to control the interferences from other elements at the standard wavelength.

**Table 2:** Instrumental and operating conditions for ICP-OES measurements

Parameter	Value
View mode	Axial
RF power (W)	1300
Nebulizer gas flow rate (L min^−1^)	0.8
Auxiliary gas flow rate (L min^−1^)	0.2
Plasma gas flow rate (L min^−1^)	15
Sample flow rate (mL min^−1^)	1.5
Standard Wavelength (nm)	238.204
Auxiliary wavelength (nm)	239.562

Before a routine analysis can be made, the instrument must be calibrated, and background corrections must be entered into the analytical program. The following six steps describe the typical procedure to execute a single analytical run using the ICP spectrometer: (1) start the torch; pump MilliQ water into the spray chamber for at least 30 min; (2) initialize the program and wait for a stable signal; (3) nebulize the most diluted standard for at least 1 min; initiate a calibration sequence with the rest of the standards from the most diluted to the most concentrated; (4) load autosampler rack with sample and standard solutions to be analyzed; (5) program a sequence to measure a verification standard with a [Fe] similar to the [Fe] of the samples every five samples; and (6) start the data collection and storage function on the computer interfaced to the spectrometer.

### Measurement of iron content by spectrophotometry

The method used in this work is based on the complexation of Fe^2+^ with *o*-phenanthroline due to the high absorptivity of the orange-red complex ion (ferrous tris-*o*-phenanthroline) formed at 510 nm. The spectrophotometric quantification of the iron concentration was carried out using a Beckman DU®530 U*V*/VIS spectrophotometer. A typical measurement, including the Fe^3+^ reduction and complexation step, is performed as follows: 200 μL (pipetted with a 20–200 μL variable volume Eppendorf research® pipette) of each standard, 200 μL MilliQ water as blank, and three times 200 μL of each dissolved sample was pipetted in 1 mL polystyrol cuvettes (LLG-cuvettes, semi-micro, PS, LLG Labware, Germany). Then, 100 μL (pipetted with a 10–100 μL variable volume Eppendorf research® pipette) 10% hydroxylamine hydrochloride solution was added, followed by the addition of 700 μL (pipetted with a 100–1000 μL variable volume Eppendorf research® pipette) 0.1% *o*-phenanthroline solution. The cuvettes were stored for 30 min in the dark to allow for complete complex formation. Then, the absorption of each standard and sample were measured at 510 nm versus the blank. For analysis of the absorption data, the absorbance of each iron standard was plotted against its concentration and a linear equation was fitted to these data points according to the Lambert-Beer-law. Then, the iron concentration of each measured sample was calculated by the use of this equation.

### Inter-institutional study

Samples were analyzed in two different institutions (ICMM/CSIC-Spain and nanoPET Pharma GmbH-Germany) by two quantification methods (ICP-OES at CSIC and spectrophotometry at nanoPET) according to the procedures (sample preparation as well as iron detection/ quantification step) established at the respective institute. Cross-validation experiments were also undertaken to analyze the uncertainty arising from the sample preparation step. Thus, the digested and diluted samples were not only subjected to the quantification step at the respective institute but also shipped to the other institute for the actual quantification step.

### Data analysis

The arithmetic mean and standard deviation values (SD) of each ICP or spectrophotometric analysis were calculated from the six concentration values (three measurement repetitions of two different dissolutions) while the percentage standard deviation (SD%) represents the value of the standard deviation with respect to the mean value. In addition, the average uncertainty of the value from each analysis was calculated as the modulus of the difference between the measured value (from each analysis at nanoPET or CSIC) and the mean value over all analyses for each particular sample.

## Results and discussion

### Influence of the protocol for sample dissolution

The combined standard uncertainty arising from the sample preparation step can be calculated by means of the root sum of the squares method using the standard individual uncertainties associated with the various stages of the sample digestion and dilution, e.g., pipetting and reading the meniscus of the volumetric flask. The uncertainties arising from the equipment (pipettes and volumetric flasks) were obtained from the calibration certificate given by the supplier (Table 3). From Table 3, we can conclude that the dilution uncertainty associated to the equipment decreases drastically when increasing the pipetting volume and the final dilution volume (determined by the volumetric flask).

**Table 3:** Combined standard uncertainties arising from the equipment (volumetric flasks and pipettes) used in the preparation of the solution, that is digestion and dilution, calculated using the rule of the root sum of the squares [28] from the standard uncertainties of fixed volume certified Eppendorf Research Plus® pipettes [29] as well as uncertainties of batch-certified DURAN® volumetric flasks

			Volumetric flask		
			5 mL	25 mL	50 mL
			0.2% ± 0.010 mL	0.06% ± 0.015 mL	0.04% ± 0.020 mL
Pipette	10 μL	0.55% ± 0.055 μL	0.57% 500 ± 2.8	0.53% 2500 ± 13	0.53% 5000 ± 26
	20 μL	0.17% ± 0.034 μL	0.26% 250 ± 0.66	0.18% 1250 ± 2.3	0.17% 2500 ± 4.4

**Fig. 2:**
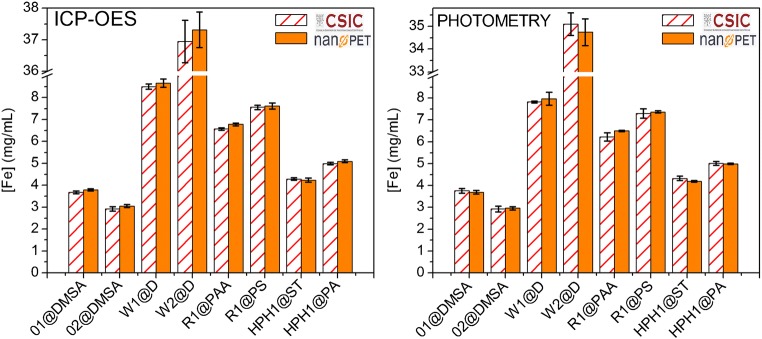
Influence of the sample preparation step made at CSIC (orange/white striped bars) and nanoPET (orange bars) for the various samples measured by ICP-OES at CSIC and by photometry at nanoPET. The error bars represent the standard deviation of the iron concentration

Uncertainties that are less than 20% of the highest component uncertainty have little impact on the overall uncertainty and can be omitted from calculation [30]. Thus, we will disregard negligible uncertainties arising from possible impurities in the commercial HCl and the distilled or MilliQ water. Therefore, in principle, we did not expect that the equipment employed in the preparation of the sample prior to the analysis could influence the uncertainty of the measurements. The results of the measurements made by ICP-OES and by spectrophotometry are presented in Fig. 2. Table 4 presents the mean average values of all measurements and the uncertainties of all the analysis.

**Table 4:** Mean average value of the iron concentration measured by both methods and uncertainties of the analysis of the samples prepared at both nanoPET and CSIC and analyzed by photometry at nanoPET and by ICP-OES at CSIC

Sample	Mean value of all measurements (mg/mL iron)	Uncertainty (%)			
		Sample preparation		Sample preparation	
		nanoPET	CSIC	nanoPET	CSIC
		Spectrophotometry at nanoPET		ICP-OES at CSIC	
O1@DMSA	3.73	0.9	1.6	0.8	1.4
O2@DMSA	2.96	0.0	2.9	1.4	1.5
W1@D	8.24	3.3	5.1	5.0	3.2
W2@D	36.03	3.5	3.6	2.6	2.5
R1@PAA	6.52	0.3	4.0	4.5	0.7
R1@PS	7.46	1.2	2.1	2.2	1.2
HPH1@ST	4.26	1.6	0.6	1.6	0.7
HPH1@PA	5.02	0.7	1.4	0.1	0.7
Average uncertainty		1.4	2.7	2.3	1.5

The Fe concentration average values obtained from the solutions at CSIC or at nanoPET are within the uncertainty of the analysis for most samples irrespective of the analytical method and the institution where the determination of iron was made (Fig. 2). Only in the case of R1@PAA, measured by ICP-OES, there is no overlapping between the values obtained from nanoPET and CSIC solutions; this was made obvious in this case due to the high precision of this particular analysis. If we compare the uncertainty percentage, the average differences are 2.3% for ICP-OES at CSIC and 2.7% for spectrophotometry at nanoPET (Table 4). This allows to conclude that ICP and spectrophotometry average results present differences lower than 3%, independently on where the samples were prepared (i.e., digested and diluted) as long as the sample preparation step was harmonized successfully. Interestingly, this value is reduced to half (1.4% for CSIC and 1.5% for nanoPET) when the sample preparation and the analysis were done in the same institution (see Table 4, first and last column).

**Fig. 3:**
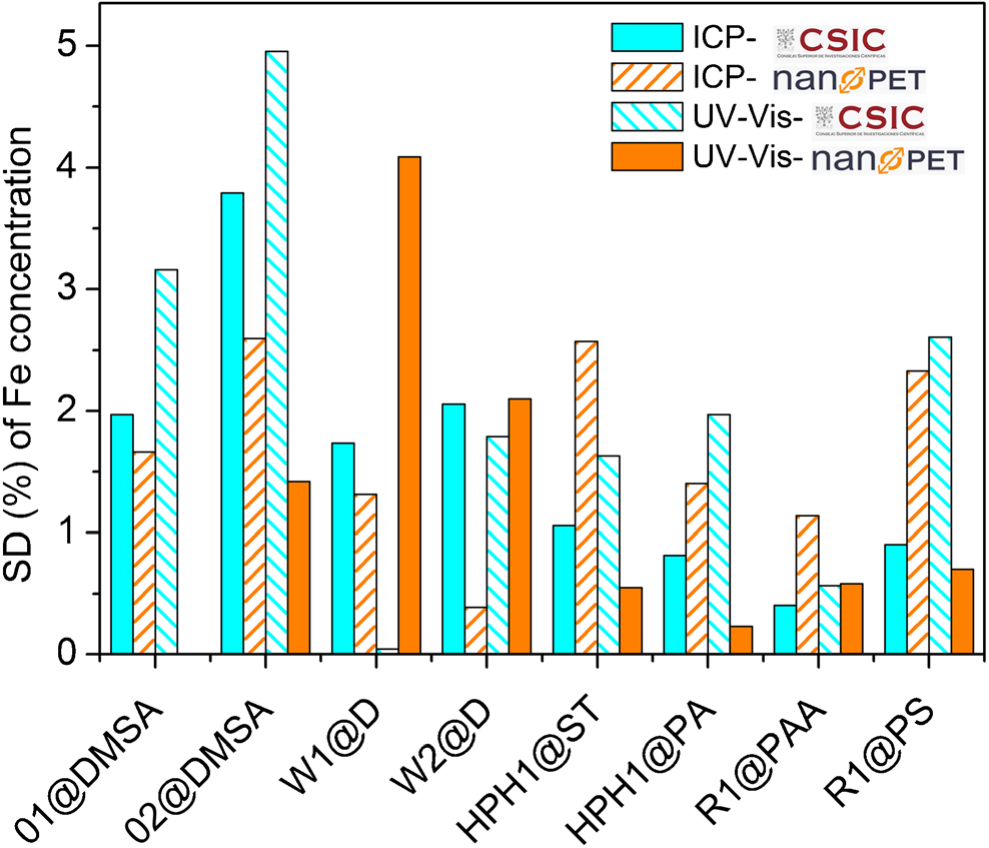
Comparison of the standard deviations (%) of the iron concentrations values of all the samples prepared and measured by ICP at CSIC (blue), prepared and measured by spectrophotometry at nanoPET (orange), prepared at CSIC and measured by spectrophotometry at nanoPET (blue stripes), and prepared at nanoPET and measured by ICP at CSIC (orange stripes)

Standard deviations for all the samples dissolved at CSIC and nanoPET and analyzed by ICP and photometry were calculated and presented in Fig. 3. The results show that the standard deviation of the samples prepared (digested/diluted) and measured at different institutions tend to be larger, but differences in behavior among the samples become clearer. This is especially pronounced in samples O2@DMSA, R1@PAA and R1@PS.

Presumably, the shipment conditions such as uncontrolled temperature and leaking of the tubes or partial evaporation affect the accuracy of the measurement. There may be other sources of uncertainty as the adsorption of iron on the container during the time mediated between the dissolution and the analysis (especially in the non-insulated cargo area during the air-shipping) or the formation of large hydrated iron compounds in solution with time. For this reason, as well as the easier practicability, we recommend carrying out the digestion of the sample in the same place where the analysis is conducted, which is also the preferred way of sample handling following good laboratory praxis (GLP). For samples prepared and measured at the same institute, we found SDs of 2.5% or below with two exceptions out of 16 analysis runs, namely O2@DMSA (SD 3.4%; prepared and measured by CSIC) and W1@D (SD 3.7%; prepared and measured at nanoPET).

### Influence of the procedure for iron determination

Values of percent difference (Δ%) (Eq. 1) [31] were used for the comparison of the analysis methods, where *C*_ICP_ is the concentration determined by ICP and *C*_photo_ is the concentration determined by spectrophotometry. The maximum value of this function is ± 200%, while a Δ% value of 0 denotes a perfect match of the analytical values and a value approaching ± 200% means there is no similarity between the values obtained by the two different analytical techniques [31]. A positive Δ% value indicates that the [Fe] determined by ICP is larger than the concentration measured by spectrophotometry.

1 $$ \Delta  \%=\frac{\left({C}_{\mathrm{ICP}}-{C}_{\mathrm{photo}}\right)\times 100}{\left({C}_{\mathrm{ICP}}+{C}_{\mathrm{photo}}\right)\div 2} $$

**Fig. 4:**
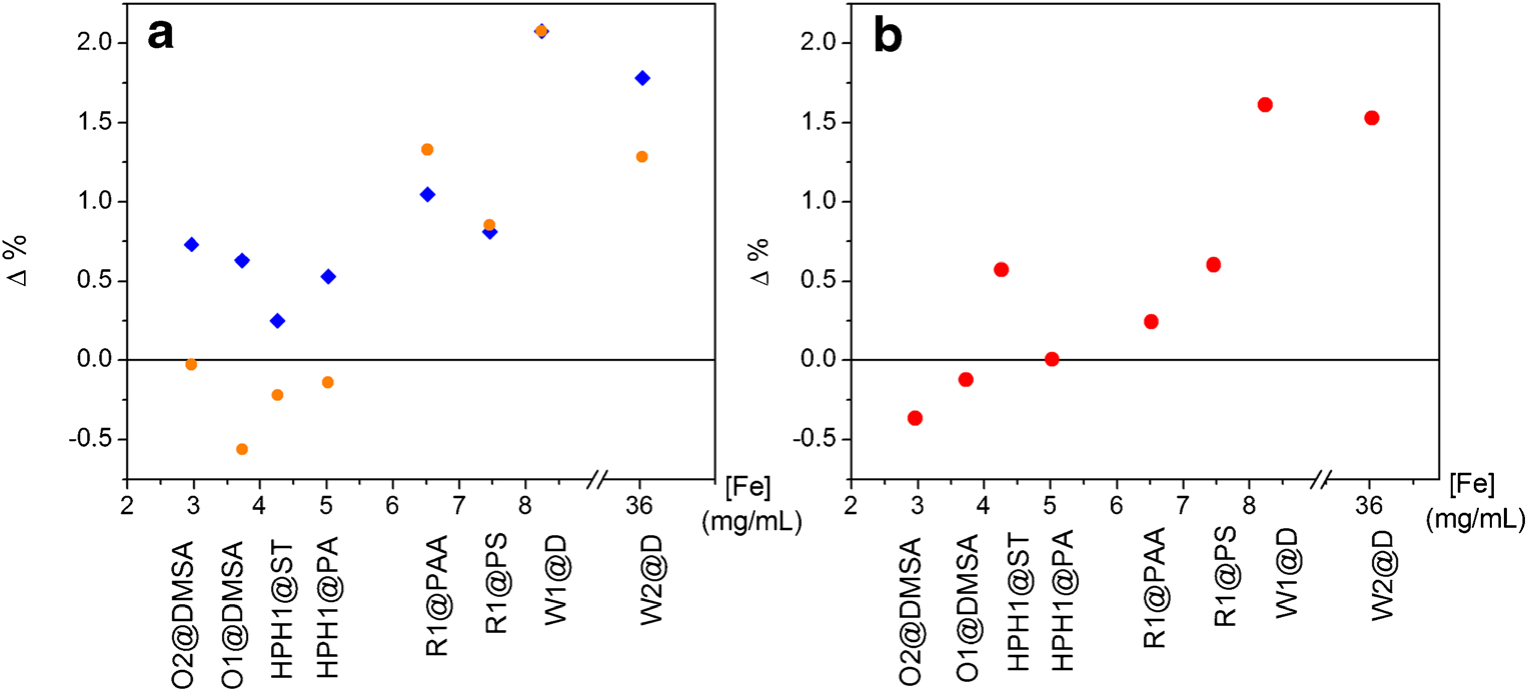
Modified Bland-Altman plot: percent difference (Δ%) between ICP and photometry as a function of the mean iron concentration (mg/mL). The name of each sample has been written in the *x* axis to ease the comparison. **a** All samples, prepared at CSIC (blue diamonds) and prepared at nanoPET (orange circles). **b** The same for samples prepared and analyzed in the same institution (prepared at CSIC and analyzed by ICP and prepared at nanoPET and analyzed by spectrophotometry)

A representation of Δ% for all the samples is shown in Fig. 4 by a modified Bland-Altman plot where we represent the percent difference (Δ%) as a function of the sample concentration.

Figure 4a represents the Δ% versus the iron concentration to determine the agreement between ICP and spectrophotometry for all the samples prepared. There are minor differences within each sample depending on the dissolution institution. The percent difference results obtained for all the samples dissolved and analyzed in the same institution are shown in Fig. 4b. The agreement among the two methods increases when the samples are prepared and analyzed in the same institution in a similar way than the reduction of the uncertainty. We conclude that part of the disagreement present in Fig. 4a comes from the preparation of the sample and to evaluate the concentration dependence of the agreement between the iron analysis made using ICP-OES and photometry is better to use the plot presented in Fig. 4b where dissolution and analysis were made at the same institution.

We can clearly see that the samples exhibiting larger percent difference values are those with larger iron concentration, that is over 8 mg/mL (W1@D and W2@D), whereas all the other samples show Δ% values around ± 0.5 which could be defined as limit of agreement for the samples with iron concentrations around 5 mg/mL. This could be attributed to the fact that pipettes are calibrated with distilled water at 20 °C. Therefore, solutions which differ greatly from water in terms of their physical properties, or temperature differences between the pipette, pipette tip, and liquid, can result in incorrect dispensing volumes [29]. Moreover, the viscosity and surface tension of colloidal suspensions of magnetic nanoparticles differ significantly from those of water especially in case of high concentrations and thus, the pipetting error may be considerably larger, especially for samples at high concentrations. Matrix effect (direct or indirect alteration or interference in response due to the presence of unintended analytes or other interfering substances in the sample) may also play a role. Even that these matrix effects were not observed in our study, eventually they could appear in similar samples. In any case, undissolved material needs to be removed (i.e., by filtration or centrifugation) prior to the analysis (especially for the ICP-OES device, whose nozzle may be collapsed). If a sol is present, the only way to decrease this source of uncertainty is to formulate a control solution with all the constituents of the sample except for the iron oxide, and prepare the standards for the calibration curves both for ICP-OES and spectrophotometry with this solution. This is not always easy, and uncertainty may increase dramatically due to this type of matrix effects in such cases.

## Conclusions and recommendations

We have successfully harmonized the iron concentration quantification procedures between CSIC (ICP-OES) and nanoPET (spectrophotometry). The uncertainty values of the iron concentration from either spectrophotometry or ICP-OES were about 1.5% on average, when samples were prepared and analyzed in the same institution. Uncertainty values around 2.5–3%, were found only for the two samples with very high iron concentrations (> 8 mg/mL).

Provided that samples have iron concentrations of about 5 mg/mL or lower, the Bland-Altman plot proves an excellent agreement of the results of the analysis methods with percent differences (Δ%) smaller than ± 0.5 and relative uncertainty values smaller than 1.5%. This value is the lower limit of uncertainty in the iron determination in magnetic nanoparticles aqueous dispersions. This uncertainty will affect the uncertainties of relevant physical properties of such colloids with respect to their biomedical applications.

Based on the findings of our study, we compiled a list of recommendations for the sample preparation step for iron concentration quantification of iron oxide nanoparticle suspensions:

1. Uncertainties arising from the pipettes and volumetric flasks decreases drastically when increasing the pipetting volume and the final volume (determined by the volumetric flask). We recommend the use of volumetric flasks of 25 or 50 mL and pipettes of minimum 10 μL (preferably 20 μL).
2. Pipettes are calibrated with distilled water at 20 °C. Therefore, solutions which differ significantly from water in terms of their physical properties, like concentrated magnetic nanoparticle suspensions, can result in incorrect dispensing volumes. For this reason, we recommend to preferably process samples of low or intermediate concentrations at about 5 mg Fe/mL rather than very high concentrations whenever possible to minimize pipetting errors.
3. Magnetic particle’s coating materials or other sample components may interfere with the measurements leading to errors in the iron concentration determination. If feasible, the manufacturer should be asked to provide a control sample with all the components of the sample except for the magnetic particles. In the case of the ICP-OES measurements, the routinely use of an auxiliary measurement wavelength is recommended to minimize and detect possible interferences from other elements present in the sample.
4. Digestion (and dilution) of the nanoparticles and measurement of the iron concentration should be accomplished in the same institution.
5. Several replications of each measurement and several dilutions (at least two) of each sample should be performed. Cross-validated and duplicated measurements help to reduce the uncertainty and to detect possible errors in the iron concentration analysis procedure of the samples. If available, a reference iron oxide nanoparticle suspension sample of known concentration should be used as an additional control during each analysis run.
